# Perceived Stress, Burnout, Professional Quality of Life, and Occupational Balance Among University Faculty in Health Sciences Disciplines in Spain—Protocol and Descriptive Results

**DOI:** 10.3390/healthcare14040494

**Published:** 2026-02-14

**Authors:** Mª Pilar Rodríguez-Pérez, Sandra León-Herrera, Angela Asensio-Martínez, Cristina García-Bravo, Sara García-Bravo, Raquel Gómez-Bravo, Elisabet Huertas-Hoyas

**Affiliations:** 1 Department of Physical Therapy, Occupational Therapy, Physical Medicine and Rehabilitation of Rey Juan Carlos University, 28922 Alcorcón, Spain; pilar.rodriguez@urjc.es (M.P.R.-P.); cristina.bravo@urjc.es (C.G.-B.); sara.garcia.bravo@urjc.es (S.G.-B.); elisabet.huertas@urjc.es (E.H.-H.); 2 Research Group Participation, Roles, Occupations and Activities for the Transformation of Communities, Rey Juan Carlos University, 28922 Alcorcón, Spain; 3 Department of Psychology and Sociology, University of Zaragoza, 50009 Zaragoza, Spain; sleon@unizar.es; 4 Institute for Health Research Aragón (IIS Aragón), 50009 Zaragoza, Spain; 5 Research Network on Chronicity, Primary Care and Health Promotion (RICAPPS, RD24/0005/0004), Carlos III Health Institute, 28029 Madrid, Spain; 6 Research Group of Humanities and Qualitative Research in Health Science (Hum&QRinHS) Universidad Rey Juan Carlos, 28922 Alcorcón, Spain; 7 Centre Hospitalier Neuro-Psychiatrique (CHNP), 9012 Ettelbruck, Luxembourg; raquelgomezbravo@gmail.com

**Keywords:** perceived stress, burnout, professional quality of life, occupational balance, university faculty

## Abstract

**Background/Objectives**: University faculty in health sciences are an underexplored population despite facing significant emotional, occupational, and organizational demands due to their dual role as educators and health professionals. These demands may increase vulnerability to perceived stress, burnout, and reduced professional quality of life. Although previous research has primarily focused on stress and burnout, evidence integrating occupational balance and personal resources, such as sense of coherence, from an occupational health perspective remains limited. This study aimed to examine perceived stress, professional quality of life, occupational balance, and satisfaction with meaningful occupations among health sciences faculty in Spain, and to analyze their associations with individual, occupational, and organizational factors within an occupation-centered and salutogenic framework. **Methods**: A cross-sectional observational study following STROBE guidelines was conducted with 253 health sciences faculty members from Spanish universities. Data were collected through an anonymous online questionnaire including validated instruments (PSS-10, OBQ-E, ProQoL, SOC-13) and items on occupational satisfaction and perceived institutional support. Descriptive statistics, *t* tests, one-way ANOVA, and Pearson correlation analyses were performed. **Results**: Participants reported moderate levels of perceived stress and occupational balance, high overall professional quality of life satisfaction, and moderate levels of compassion fatigue. Higher perceived stress scores were observed among women and younger faculty members. Occupational balance, burnout, and satisfaction measures showed significant differences according to age and years of teaching experience. Perceived institutional support differed across organizational domains, academic positions, and types of institution. **Conclusions**: Health sciences faculty in Spain experience notable psychosocial demands affecting stress, occupational balance, and professional quality of life. Occupational balance and sense of coherence emerged as relevant constructs associated with lower perceived stress and burnout and higher professional satisfaction. By integrating these occupation-centered and salutogenic resources, the study extends existing research beyond traditional stress–burnout models and contributes to a more comprehensive understanding of professional well-being among health sciences faculty.

## 1. Introduction

In recent years, the emotional well-being of healthcare professionals has attracted increasing attention in the scientific literature. Although numerous studies have focused on clinical workers and students, university faculty in health sciences disciplines remain a comparatively underexplored group, despite facing significant emotional demands associated with their dual role as educators and professionals in high-pressure environments [1].

Available evidence indicates that university faculty are particularly vulnerable to elevated psychosocial risks, including stress and emotional exhaustion, largely due to heavy workloads, limited of institutional support, and performance-related academic pressures [2].

Recent studies conducted in the Spanish context support and extend these findings. In a sample of 1560 university professors, 20.8% exhibited high levels of emotional exhaustion, 5.3% showed elevated depersonalization, and approximately half reported low levels of personal accomplishment. Furthermore, strong associations were observed between burnout dimensions, stress, depression, and poorer physical and mental quality of life [3]. Among health sciences faculty, these challenges may be further intensified by the additional responsibility of guiding students in complex and emotionally demanding clinical settings, while maintaining high professional standards [4]. These demands are compounded by the need to balance teaching responsibilities with research requirements, including accreditation processes, publications in high-impact journals, competitive funding acquisition, and increasing teaching workloads, all of which are associated with elevated stress, emotional exhaustion, and job dissatisfaction [4,5].

From a gender perspective, research has consistently shown that gender plays a significant role in the organization of academic work and professional experiences within universities [6]. Women are often overrepresented in health sciences disciplines and are more likely to assume multiple academic roles, including teaching, research, mentoring, student support, and administrative duties. This multiplicity of responsibilities substantially increases workload and may adversely affect emotional well-being and occupational balance [7,8]. Consistent with these findings, studies conducted in Spain indicate that female faculty members report higher levels of perceived stress, emotional exhaustion, and burnout-related physical symptoms than their male counterparts [9]. These patterns are further reinforced by institutional characteristics, such as high disciplinary diversity, extensive teaching experience, and frequent role accumulation, which have been linked to increased professional stress and burnout, particularly among women [10].

The combined demands of teaching, research and clinical practice create an especially challenging professional context for health sciences faculty. This convergence places them in a situation comparable to other care-oriented professions, such as medicine, nursing, psychology, and education, in which burnout has been extensively documented [11,12]. Prolonged exposure to institutional pressures, high emotional demands, and strong professional commitment substantially increases the risk of anxiety, depression, and burnout, thereby compromising both quality of work life and engagement in teaching activities [13,14,15].

Despite extensive research on stress and burnout among university faculty, studies adopting an integrative occupational perspective remain limited, particularly in relation to health sciences academics. Specifically, the literature reveals limited exploration of how these demands affect key dimensions of daily functioning, such as daily routines, work–life balance, autonomy, sense of competence, and the balance between external demands and personal resources [16,17].

Within this framework, occupational balance has emerged as a central determinant of health and well-being. It refers to the subjective perception of having an appropriate and meaningful distribution of daily activities [18]. In occupational health research, this concept provides a valuable framework for examining how academic, professional, and personal demands are integrated into daily life. More specifically, it facilitates the identification of situations in which work-related demands exceed available personal resources, generating sustained tensions that may lead to chronic stress, professional burnout, and health deterioration. Recent studies among health science students have demonstrated associations between occupational balance, healthy lifestyles, resource availability, and academic environmental factors [16]. Additionally, research in Spanish university populations suggests that academic overload and ineffective time management contribute to occupational imbalance and increased psychological distress [19].

The sense of coherence (SOC) also plays a crucial role in stress management and health maintenance by shaping how individuals perceive and cope with adverse situations. Within psychosocial work models, such as the Job Demands–Resources framework, SOC is recognized as a key factor in stress regulation, burnout prevention, and sustained work engagement, particularly among individuals with mental health issues [20]. Moreover, work-related resources, including autonomy and social support, have been shown to strength work-specific sense of coherence, generating positive resource gain cycles and sustained improvements for occupational health [21]. Nevertheless, research examining SOC among university faculty remains limited, particularly regarding its interaction with stress, burnout, and occupational balance in the context of health sciences [22].

During the COVID-19 pandemic, substantial disruptions in occupational balance and quality of life were observed among university staff, particularly among those who had contracted the virus. These changes were reflected in lower professional satisfaction and higher rates of burnout, highlighting faculty vulnerability during the health crisis [15,23]. However, existing research has addressed the impact of this situation in a fragmented manner, with limited integration of occupational health constructs, such as occupational balance and sense of coherence, into comprehensive analyses of stress and burnout among health sciences faculty from a preventive and occupational perspective [15].

Given this context, the present study aims to analyze the relationships between perceived stress, occupational balance, satisfaction with meaningful occupations, and professional quality of life among health sciences faculty in Spanish universities. The study integrates psychosocial constructs that have traditionally been examined separately, such as stress, burnout syndrome, and well-being, with key occupational health variables, including occupational balance and sense of coherence within the health sciences academic context.

In addition, the study assesses the contribution of individual, occupational, and organizational factors, including perceived institutional support, to provide a more comprehensive understanding of their role in shaping professional well-being. Through this integrative approach, the study seeks to advance empirical knowledge on the mechanisms through which academic and clinical demands affect faculty health and to provide a solid methodological foundation for future comparative research in the European context.

## 2. Materials and Methods

### 2.1. Design

The study was conducted using a cross-sectional observational design. To ensure methodological rigor and transparency in reporting the results, the study’s design, analysis, and reporting were guided by the recommendations of the Strengthening of the Reporting of Observational Studies in Epidemiology (STROBE) guidelines for observational studies (Table S1) [24].

The design and conduct of the study adhered to the ethical principles of the Declaration of Helsinki and to applicable data protection regulations. The research protocol was reviewed and approved by the Ethics Committee of Universidad Rey Juan Carlos, Spain (Approval Code: 070720255722025), and all participants provided informed consent prior to completing the questionnaire.

### 2.2. Sample

The target population of the study consisted of undergraduate and postgraduate university faculty in health science disciplines at Spanish and European universities. These disciplines included Medicine, Nursing, Physiotherapy, Occupational Therapy, Psychology, Dentistry, and Pharmacy, thereby reflecting the academic diversity characteristic of the health field. Data collection for the national phase of the study was conducted between July and November 2025.

To ensure the relevance and suitability of the participant profile, specific eligibility criteria were established. Inclusion criteria were affiliation at the time of the study with a university program in health sciences; having a minimum teaching experience of six months; and participating voluntarily. Exclusion criteria were administrative or management staff without direct teaching responsibilities; faculty on extended leave during the data collection period; and faculty based outside the European Union.

A non-probabilistic sampling approach was employed, combining convenience and snowball sampling techniques, consistent with the exploratory nature of the study and the need to reach faculty from different institutions and programs. Health sciences university faculty represent a highly heterogeneous population in terms of disciplinary background, academic rank, contractual conditions, and institutional affiliation, and no comprehensive centralized sampling frame is currently available at the national level. In this context, convenience sampling facilitated initial access to eligible participants, while snowball sampling enabled the recruitment of faculty from diverse universities and academic profiles.

Participant recruitment was carried out through invitations sent via institutional email and distributed through the corresponding faculties. Each invitation included a link to the digital questionnaire, the study information sheet, and the informed consent form. This recruitment strategy is widely used in occupational and academic health research when studying hard-to-reach professional populations and allows for the generation of preliminary evidence to inform future, more controlled studies.

The study comprises two successive phases:

1. National phase (Spain): initial data collection, the preliminary descriptive results of which are presented in this article.
2. European phase: planned as an extension of the same protocol to compare trends between Spanish and European faculty.
3. The sample size was determined using standardized procedures with G*Power 3.1.9.4 software, taking into account the analytical objectives of each phase of the study.

#### 2.2.1. Phase 1: National Sample (Spain)

To conduct exploratory comparisons between different faculty subgroups, such as academic discipline or employment contract type, a one-way ANOVA model was employed (F tests: ANOVA—fixed effects, omnibus, one-way). For the calculation of the sample size for the national phase, the required statistical parameters were predefined in accordance with recommendations for observational studies in the social sciences. A significance level of 0.05, a statistical power of 80%, and a medium effect size (f = 0.25) were established, following Cohen’s guidelines. In addition, six comparison groups were assumed, corresponding to the main faculty categories or subgroups considered in the exploratory analysis.

Under these parameters, the minimum required sample size was 216 participants. To account for potential attrition or incomplete questionnaires, a 10% increase was applied, resulting in a recommended minimum sample size of 237 participants for the national phase.

A total of 253 questionnaires were collected and included in the analysis. All questionnaires met the predefined inclusion criteria and contained complete data for the main study variables; therefore, no responses were excluded due to missing, invalid, or inconsistent information.

Due to the open recruitment strategy, which combined institutional email dissemination and snowball sampling, the exact number of faculty members who received the invitation could not be determined. As a result, a precise response rate could not be calculated. This limitation is inherent to the sampling approach; however, it enabled broad participation across institutions and academic profiles, consistent with the exploratory aims of this national phase.

#### 2.2.2. Phase 2: European Sample

For comparisons between two groups (Spain vs. other European countries), an independent-sample Student’s *t* test will be used (means: difference between two independent means, two groups).

For this future phase of the study, standard statistical parameters were defined, including a significance level of α = 0.05, a statistical power of 80% (1 − β = 0.80), and a medium effect size (d = 0.50), in accordance with methodological recommendations for comparative research in the social sciences. Under these conditions, the minimum required sample size was 102 participants (51 per group). Allowing for an additional 10% to compensate for potential losses, a minimum sample size of 112 participants was estimated.

### 2.3. Variables and Instruments

Data were collected using a structured, self-administered online questionnaire developed in Microsoft Forms^®^, which ensured anonymity and facilitated participation from faculty across Spanish universities. Dependent variables included perceived stress, professional quality of life, occupational balance, and satisfaction with meaningful occupations. Independent variables comprised sociodemographic and occupational characteristics, sense of coherence, and perceived institutional support.

Perceived stress was measured using the Spanish-validated Perceived Stress Scale (PSS-10) by Remor (2006) [25], which demonstrates adequate psychometric properties (α = 0.82). The scale conceptualizes perceived stress as the extent to which individuals evaluate situations in their lives as stressful. The scale consists of 10 items, using a 5-point Likert response format ranging from never (0) to very often (4). The total score is obtained by summing the items, with a possible range from 0 to 40, where higher scores indicate greater levels of perceived stress. In the present sample, the scale demonstrated excellent internal consistency (α = 0.90). The PSS-10 has been widely used in academic and health-related populations, including university faculty and healthcare professionals, showing robust psychometric performance in occupational and educational contexts [13].

Occupational balance was assessed using the Spanish version of the Occupational Balance Questionnaire (OBQ-E) [26], which conceptualizes occupational balance as a perceived sense of harmony among daily occupations and a necessary condition for enabling occupations to effectively contribute to health and well-being. This instrument consists of 13 items, with a 6-point Likert-type response scale (0 = “strongly disagree” to 5 = “strongly agree”). The total possible score ranges from 0 to 65, with higher scores indicating greater occupational balance. It demonstrates adequate internal consistency (α = 0.87) and excellent reliability in the present sample (α = 0.94). Recent studies support the applicability of the OBQ-E to health sciences students and professionals, reinforcing its relevance for occupation-centered and occupational health research [17].

Additionally, items adapted from the Canadian Occupational Performance Measure (COPM) [27] were used to assess occupational satisfaction with work-related activities, interpersonal relationships, and perceived work–rest–self-care balance, that is, the extent to which individuals experience fulfillment and positive emotional appraisal in these domains.

Professional quality of life was evaluated using the Spanish-validated Professional Quality of Life Scale (ProQoL) [28], assessing compassion satisfaction (α = 0.89), compassion fatigue (α = 0.84), and burnout (α = 0.76), with excellent internal consistency in this study (α = 0.86). The scale comprises 30 items and is administered using a five-point Likert format (1 to 5), with higher scores indicating greater intensity of the assessed dimension. The ProQoL has been extensively validated in healthcare and academic-related populations, allowing for a differentiated assessment of compassion satisfaction, burnout, and compassion fatigue [12]. In accordance with the ProQoL theoretical framework, burnout and compassion fatigue were operationalized and interpreted as distinct constructs. Burnout was considered a work-related syndrome reflecting emotional exhaustion, depersonalization, and reduced professional efficacy resulting from chronic occupational stress, whereas compassion fatigue (secondary traumatic stress) was conceptualized as emotional distress associated with indirect exposure to others’ suffering. Although conceptual overlap between these constructs has been discussed in the literature, the ProQoL allows for their differentiated assessment, which was maintained in the present study to ensure conceptual and analytical clarity.

Sense of coherence, defined as the degree of individuals’ confidence that the internal and external stimuli in their lives are comprehensible, coherent, and predictable, was measured using the Spanish version of the Sense of Coherence-13 scale (SOC-13) [29], which assesses comprehensibility, manageability, and meaningfulness, and has shown adequate psychometric properties (α = 0.80). The scale consists of 13 items rated on a 7-point Likert-type scale, with response options ranging from very often (1) to rarely or never (7). The total score is obtained by summing the responses to the 13 items, with higher scores indicating a greater sense of coherence. In the present sample, the scale demonstrated excellent internal consistency (α = 0.90). The SOC-13 has shown adequate validity in academic and health-related populations, supporting its use as a key personal resource in occupational health research [22].

Perceived institutional support, understood as individuals’ subjective perception that their organization values their contributions, is concerned about their well-being, and offers the resources, guidance, and support necessary to promote successful performance, was explored through 10 ad hoc items addressing key organizational dimensions (e.g., well-being, work–life balance, autonomy, resources, and recognition). As these items do not form a validated scale, each item was analyzed independently. All items were rated on a five-point Likert scale (1 = strongly disagree; 5 = strongly agree). All instruments employed in this study use Likert-type response formats, which are widely accepted in health sciences and educational research. Consistent with methodological recommendations, Likert-scale data were treated as continuous variables, allowing for the application of parametric statistical analyses when distributional assumptions were met [30]. The development of these ad hoc items was conceptually informed by prior literature on occupational health, perceived organizational support, and academic work environments, as well as by the authors’ clinical and research experience in university and healthcare settings. Given the exploratory nature of this phase, the items were designed to capture key organizational dimensions rather than to constitute a validated scale; therefore, each item was analyzed independently to provide an initial descriptive assessment of perceived institutional support.

Sociodemographic and professional variables were collected using an ad hoc questionnaire and included age, gender, marital status, number of children, type of employment contract, academic position, type of institution, disciplinary area, and years of teaching experience.

### 2.4. Data Analysis

Quantitative analysis was conducted using IBM SPSS Statistics software (version 31).

Prior to inferential analyses, the distribution of continuous variables was examined using the Shapiro–Wilk test to assess normality. As the assumptions for parametric analyses were met, parametric statistical tests were applied. Subsequently, a detailed description of the sociodemographic and occupational characteristics of the sample, as well as the main study variables, was performed using descriptive statistics (frequencies and percentages for categorical variables and means and standard deviations for continuous variables).

To analyze differences between groups according to categorical sociodemographic and professional variables with more than two categories (e.g., academic position, marital status, or years of teaching experience), one-way analyses of variance (ANOVA) were performed. For group comparisons involving two categories, independent-sample *t* tests were applied when appropriate. Effect sizes were calculated and reported as Cohen’s *d* for two-group comparisons and eta squared (η^2^) for ANOVA analyses.

Additionally, Pearson correlation coefficients were computed to examine the relationships between continuous variables, specifically between age and scores on the perceived stress, professional quality of life, occupational balance, sense of coherence, perceived institutional support, and occupational satisfaction scales. For correlation analyses, effect sizes were reported using Pearson’s *r* and the coefficient of determination (*r^2^*). For perceived institutional support, analyses were conducted at the item level, as no aggregated score was calculated. In all analyses, a statistical significance level of *p* < 0.05 was set. Only statistically significant associations are reported in the Results section and corresponding tables, while complete results of all conducted analyses, including non-significant findings, are provided in the Supplementary Materials.

Given the exploratory nature of this national phase, no formal correction for multiple comparisons was applied. This decision was made to minimize the risk of Type II errors in the context of hypothesis-generating research. Accordingly, the results are interpreted with caution, and emphasis is placed on the magnitude and direction of associations rather than solely on statistical significance.

## 3. Results

The present results correspond to the first national phase of the project and include data from university faculty in health sciences disciplines in Spain (N = 253). This phase provides a descriptive characterization of sociodemographic and occupational profiles, as well as psychosocial variables and their associations with individual and organizational factors. Findings from the planned European phase, which will enable cross-national comparisons, are not included in the present article.

### 3.1. Sociodemographic and Professional Characteristics of the Sample

The sample consisted of 253 faculty members from different disciplines and geographical regions. Women represented 67.6% of the sample, with a mean age of 46.1 years. Most participants were married and had either two children or none. The majority were employed full-time (88.1%) and worked at public universities (83%), with Associate Lecturer and Senior Lecturer being the most frequent academic positions. Nearly half of the sample (47.4%) reported more than ten years of teaching experience, indicating a well-established cohort with consolidated academic trajectories. Teaching activities were predominantly carried out at the undergraduate level, although combinations with postgraduate teaching were common. Disciplinary distribution showed greater representation from Nursing, Physiotherapy, Medicine, Occupational Therapy, and Psychology. Detailed information is provided in Table 1.

### 3.2. Descriptive Results of Psychosocial Variables

Descriptive statistics for psychosocial variables are presented in Table 2.

Faculty reported a mean perceived stress score of 15.5 ± 7.4, with nearly half of the sample (49%) falling within the moderate range. Occupational balance was also moderate (42.5 ± 12.2), while more than half of the participants showed high levels of professional quality of life satisfaction (53.4%). Compassion fatigue levels were predominantly moderate (74.3%).

Job satisfaction showed high values for performance-related activities (M = 7.6 ± 1.5) and comparatively lower values in interpersonal relationships (M = 6.1 ± 2.1) and work–rest–self-care balance (M = 5.8 ± 2.3). Sense of coherence showed moderate values (67.1 ± 11.2).

Perceived institutional support varied across domains: higher values were reported for autonomy in teaching and research and for the collaborative work climate, whereas lower values were observed in institutional concern for well-being, availability of mental health resources, and consideration of actual working conditions.

### 3.3. Associations Between Sociodemographic/Professional Variables and Psychosocial Constructs

Given the exploratory nature of the analyses, the interpretation of results emphasizes the direction and magnitude of observed effects rather than statistical significance alone, with effect sizes reported to support a contextualized interpretation. Table 3 summarizes the statistically significant associations and group differences observed, while the complete results of all analyses, including non-significant findings, are presented in the Supplementary Material.

Perceived stress showed higher values among women compared to men, although the associated effect size was small, indicating a modest magnitude of this difference (F = 3.902, *p* = 0.008, d = 0.045), and was negatively associated with age, with a small-to-moderate effect size, indicating that stress levels tended to decrease modestly as age increased (r = –0.229, *p* < 0.001, r^2^ = 0.05). Stress levels also varied by academic position (F = 2.983, *p* = 0.012, η^2^ = 0.058), with Lecturers and Permanent Lecturers reporting higher scores, and by years of teaching experience (F = 3.050, *p* = 0.018, η^2^ = 0.047), with higher scores among faculty with less or intermediate teaching experience compared to those with more than 10 years.

Occupational balance was positively associated with age (r = 0.191, *p* = 0.002, r^2^ = 0.04) and differed according to marital status (F = 3.532, *p* = 0.008, η^2^ = 0.054), academic position (F = 3.328, *p* = 0.006, η^2^ = 0.064), and years of teaching experience (F = 3.080, *p* = 0.017, η^2^ = 0.047), with small to moderate effect sizes. Men reported significantly higher occupational balance scores than women (F = 0.171, *p* = 0.020, d = 0.315). Occupational balance scores were higher among married faculty, those in the “other positions” category, and those with more than 10 years of teaching experience.

Regarding professional quality of life, compassion satisfaction varied by marital status (F = 7.174, *p* < 0.001, η^2^ = 0.104), with married faculty reporting the highest scores and cohabiting partners the lowest, and by academic position (F = 3.946, *p* = 0.002, η^2^ = 0.075), with Permanent Lecturers reporting the lowest levels, both with small to moderate effect sizes. Compassion satisfaction also differed by type of institution, with faculty working at public universities reporting higher levels than those at private institutions (F = 3.718, *p* = 0.026, η^2^ = 0.029).

Burnout showed a negative association with age, with a small effect size, suggesting a gradual and limited decrease in burnout levels with increasing age (r = –0.219, *p* < 0.001, r^2^ = 0.048), and was higher among women than among men, with a small effect size, suggesting a limited magnitude of the observed difference (F = 0.273, *p* = 0.030, d = 0.293). Secondary traumatic stress differed by marital status (F = 4.026, *p* = 0.004, η^2^ = 0.061), with higher scores in faculty in domestic partnerships.

Sense of coherence differed by academic position (F = 2.707, *p* = 0.021, η^2^ = 0.053), with Associate Lecturers showing the highest scores. Job performance satisfaction also varied by academic position (F = 5.324, *p* < 0.001, η^2^ = 0.098), with full professors and faculty in “other positions” reporting higher satisfaction. Interpersonal satisfaction was positively associated with age (r = 0.125, *p* = 0.049, r^2^ = 0.016) and differed by academic position, with senior academic ranks reporting higher interpersonal satisfaction than junior positions (F = 3.081, *p* = 0.010, η^2^ = 0.060). Satisfaction with work–rest–self-care balance was positively associated with age (r = 0.187, *p* = 0.003, r^2^ = 0.035) and differed by gender, with men reporting higher satisfaction than women (F = 0.029, *p* = 0.005, d = 0.383).

Finally, perceived institutional support differed by type of institution, with faculty at public universities reporting higher scores for institutional concern for faculty well-being (F = 3.139, *p* = 0.045, η^2^ = 0.025), work–life conciliation measures (F = 3.216, *p* = 0.042, η^2^ = 0.025), support in facing emotional or professional difficulties (F = 7.918, *p* < 0.001, η^2^ = 0.060), and opportunities to express professional concerns (F = 3.942, *p* = 0.021, η^2^ = 0.031), as well as a collaborative and respectful work climate (F = 11.941, *p* < 0.001, η^2^ = 0.088). Academic position was also associated with perceived availability of mental health resources (F = 2.268, *p* = 0.048, η^2^ = 0.044) and consideration of actual working conditions (F = 2.573, *p* = 0.027, η^2^ = 0.050), with full professors reporting higher scores. Autonomy in teaching and research tasks was positively associated with age (r = 0.152, *p* = 0.016, r^2^ = 0.023) and differed by academic position (F = 2.256, *p* = 0.050, η^2^ = 0.044), with senior academic ranks reporting greater autonomy. While statistically significant, effect sizes were generally small to moderate, indicating that differences reflect patterns of perception rather than strong practical effects.

## 4. Discussion

The present study aimed to examine professional quality of life, perceived stress, occupational balance, and satisfaction with meaningful occupations among university faculty working in health sciences disciplines in Spain, as well as to identify the individual, occupational, and organizational factors associated with these outcomes. Using a cross-sectional design, the findings provide a comprehensive characterization of the psychosocial and occupational well-being of this group, revealing differential patterns according to age, teaching experience, and academic position [31].

The results should be interpreted as descriptive associations that allow for the characterization of the psychosocial and occupational profile of university faculty in health sciences in Spain. This approach reflects the exploratory nature of the study. Although several associations reached statistical significance, effect sizes were generally small to moderate, indicating that these differences reflect descriptive patterns rather than strong practical impacts. This profile is marked by moderate levels of perceived stress, a correspondingly moderate level of occupational balance, and heterogeneous perceptions of institutional support. This pattern is consistent with recent literature highlighting the sustained exposure of this group to high emotional and occupational demands within intensive academic contexts. In this regard, previous studies have identified university faculty as a group particularly vulnerable to psychosocial risks derived from work overload, academic pressure, and multifunctional role performance [2].

Within this framework, perceived stress represents one of the central elements of faculty members’ work experience. The levels observed were within a moderate range, with significant differences according to gender, age, teaching experience, and academic position, a pattern previously described in research on academic stress and occupational health [5,10]. In particular, women reported higher levels of stress, in line with studies indicating a greater emotional and organizational burden associated with the performance of academic and relational roles that are often underrecognized or rendered invisible [7,8]. Likewise, faculty members with lower or intermediate teaching experience showed higher stress levels, which is consistent with literature identifying the early stages of the academic career as periods of heightened emotional vulnerability [11,12].

The negative association observed between age and perceived stress suggests that accumulated experience and professional stability may be associated with more adaptive responses to academic demands, as reported in studies focusing on academic trajectories and work-related stress coping processes [5,13]. Similarly, the higher stress levels identified among intermediate academic positions—such as Assistant Professor and Permanent Labor Faculty/Contracted Doctor—reinforce the idea that the early and intermediate phases of the academic career are marked by accreditation processes, continuous evaluation, and pressure for scientific productivity [4], particularly in health sciences disciplines that involve clinical supervision and a high emotional workload [12].

This pattern becomes especially relevant when considering the high female representation in the sample, a characteristic widely documented within the field of health sciences. Several studies have shown that women in academic contexts tend to assume a greater number of simultaneous academic roles, including tutoring, management, and emotional support for students, tasks that frequently receive limited institutional recognition [7,8]. The combination of female overrepresentation, multirole performance, and prolonged professional trajectories has been associated in the literature with higher levels of emotional exhaustion and occupational imbalance, particularly among women [10].

Beyond stress, the analysis of professional quality of life provides relevant insights into the emotional impact of academic work. In this study, significant differences were observed in compassion satisfaction according to marital status and academic position, as well as higher levels of compassion fatigue among younger faculty members. This finding is consistent with research describing greater emotional exposure and a less developed repertoire of coping resources during the early stages of the professional trajectory [12,14]. By contrast, the absence of significant differences in the burnout subscale suggests that, at this preliminary stage, severe emotional exhaustion does not constitute a generalized phenomenon within the sample, although differential patterns associated with contextual variables were observed, in line with recent reviews on burnout in academic and healthcare professions [11,13].

From an occupational perspective, the inclusion of occupational balance provides substantial added value to the analysis of university faculty well-being. Unlike traditional indicators focused on distress, this construct offers insight into how educators perceive the organization, meaning, and sustainability of their daily occupations. The mean occupational balance score reflected a moderate level, with notable variability across participants, a result consistent with studies describing how the multiplicity of roles and the accumulation of responsibilities are frequently linked to difficulties in the meaningful organization of daily occupations [16,17]. Previous research has indicated that university faculty in health sciences tend to present lower levels of occupational balance compared to other professional groups, due to the convergence of teaching, research, clinical, and administrative demands [15].

In the present study, occupational balance showed positive associations with age and years of teaching experience, as well as differences according to marital status and academic position. These findings are consistent with evidence suggesting that occupational balance tends to improve across the life course and professional trajectory, as individuals report greater familiarity with strategies for organizing, prioritizing, and regulating their meaningful occupations [32]. Additionally, male participants showed higher levels of occupational balance compared to female participants, which aligns with previous literature reporting gender-based disparities in the distribution of occupational roles, caregiving responsibilities, and expectations regarding work–life balance [33]. These findings reinforce the usefulness of occupational balance as a sensitive indicator for identifying areas of vulnerability and adaptation in occupational health, complementing traditional measures of stress and burnout.

Consistent with these results, satisfaction with different areas of occupational performance was also positively associated with age, particularly in the interpersonal domain and in the balance between work, rest, and self-care, further supporting the link between life stability, professional experience, and a more positive perception of occupational performance [16,17].

From the perspective of personal resources, sense of coherence differed according to academic position, which is particularly relevant within the framework of the salutogenic model. This construct allows for a better understanding of how individuals interpret work demands as comprehensible, manageable, and meaningful, which has been conceptualized as a key psychological resource in relation to stress [34].

The combined inclusion of occupational balance and sense of coherence represents a distinctive contribution of the present study, as it allows for a broader analysis of professional well-being from an occupational health perspective. Both constructs can be conceptualized as salutogenic indicators, focusing on individuals’ capacity to organize their occupational life and to give meaning to the demands of academic work.

Finally, perceived institutional support was significantly associated with university type, age, and academic position. These findings describe differentiated patterns in how faculty members perceive organizational resources, autonomy, and support across institutional and career contexts, rather than indicating institutional effects. Consistent with previous literature, organizational characteristics such as workplace climate, perceived autonomy, and access to support resources tend to co-occur with faculty members’ reported work experiences and professional trajectories [15,23]. In this regard, age and academic trajectory were associated with higher perceived autonomy and access to certain resources, which may be indicative of accumulated experience, role familiarity, and greater confidence in navigating institutional structures. Conversely, the lack of significant associations in areas such as workload and institutional recognition of teaching highlights domains in which perceptions of support remain heterogeneous across faculty groups, as previously reported in studies on academic occupational health and professional quality of life [10,15]. These findings should therefore be interpreted as indicative of perceived support patterns rather than as evidence of institutional impact.

Overall, these findings describe a highly qualified faculty body operating within a context of multiple demands, with emotional and occupational adjustment processes that appear to evolve throughout the academic career. The associations observed with age, experience, and academic position underscore the need to consider academic careers as dynamic processes in which emotional and occupational needs vary according to life stage and professional development [5,10].

From a cautious interpretation consistent with the cross-sectional design, these findings reinforce the importance of continuing to deepen the analysis of these factors in future phases of the project, incorporating inferential and comparative approaches at the European level. Likewise, the findings highlight the relevance of considering institutional strategies grounded in a salutogenic and occupation-centered approach, oriented toward supporting self-care, sustainable role distribution, improvements in work climate, and recognition of teaching work.

## 5. Conclusions

This study offers a comprehensive perspective on the professional well-being of university faculty in health sciences in Spain, outlining a profile characterized by moderate levels of perceived stress and occupational balance, together with heterogeneous perceptions of institutional support. These findings are consistent with the coexistence of high academic and organizational demands with personal and contextual resources that vary according to age, professional trajectory, and academic position.

From an interpretation consistent with the cross-sectional nature of the study, the results suggest that sociodemographic and professional factors—such as gender, age, years of teaching experience, and academic rank—are differentially associated with perceived stress, professional quality of life, occupational balance, and sense of coherence. In this regard, younger faculty members and those in intermediate stages of their academic careers tend to display, descriptively, less favorable profiles across several of these dimensions, highlighting the importance of considering academic trajectories as dynamic processes with evolving needs over time.

The trends observed in this preliminary phase provide a foundation for the development of inferential analyses in subsequent stages of the project, as well as practice-informing insights that may help guide future reflections and research on emotional well-being and occupational balance among university faculty.

This study underscores the relevance of situating university faculty well-being within contemporary frameworks of occupational health in higher education. In the Spanish context, the Spanish Strategy for Occupational Safety and Health 2023–2027 (EESST) [35] adopts a comprehensive approach to the management of occupational risks, including psychosocial risks, while actively promoting health and well-being in university settings. The Strategy emphasizes the need to anticipate risks arising from digital, demographic, and organizational transformations, as well as the importance of integrating a gender perspective and ensuring the protection of the most vulnerable groups. Within the university context, these strategic priorities are consistent with approaches that emphasize healthy work environments, the strengthening of institutional support, the improvement of work–life balance, and the fostering of organizational cultures grounded in participation, respect, and the sustainability of academic work.

### Strengths and Limitations

This study presents several strengths that support the significance and relevance of its findings within the field of occupational health among university faculty.

First, the size and diversity of the sample stand out, comprising 253 faculty members from different health sciences disciplines. This diversity provides a broad and contextualized perspective on university faculty in the health sciences, characterized by the combination of teaching, research, and, in many cases, clinical responsibilities. Moreover, the use of internationally validated psychometric instruments enhances methodological rigor and facilitates the comparison of results with previous literature.

Another strength of the study is its integrative approach, which simultaneously examines variables traditionally addressed independently, such as stress and professional quality of life, alongside constructs specific to occupational health, such as occupational balance, sense of coherence, and perceived institutional support. This approach enables a more holistic understanding of the well-being of health sciences faculty.

Finally, the project has a European expansion perspective, which in later phases will enable the exploration of differences across university contexts and the conduct of broader comparative analyses.

Nevertheless, the study also presents several limitations that should be considered. Its cross-sectional design precludes establishing causal relationships between the analyzed variables, limiting conclusions to descriptive associations. This characteristic limits the interpretation of the results and necessitates caution when inferring potential explanatory mechanisms underlying the professional well-being of faculty. Similarly, the preliminary nature of this phase, based exclusively on descriptive analyses, highlights the need to incorporate inferential analyses and multivariate models in later stages of the project to identify significant predictors.

The non-probabilistic sampling strategy may introduce self-selection bias, thereby limiting the generalizability of the findings to the broader population of university faculty in health sciences. In addition, the absence of correction for multiple comparisons may increase the risk of Type I error; therefore, the findings should be interpreted as exploratory and require confirmation in future studies employing adjusted and multivariate analytical approaches.

Additionally, the use of self-report measures may introduce biases stemming from social desirability or from the subjective perception of one’s own working and health conditions. However, the qualitative component, which will be developed subsequently, will allow for a deeper exploration of faculty experiences, perceptions, and needs from a more interpretative and contextualized perspective.

Regarding institutional support, its assessment through an ad hoc index, although demonstrating adequate internal consistency, limits direct comparability with other studies and should be considered exploratory at this stage. In this context, future studies should incorporate longitudinal designs and multivariate analyses to examine more complex relationships among demands, personal and contextual resources, and well-being outcomes. The validation of the institutional support instrument and the inclusion of qualitative methodologies would contribute to a deeper and more contextualized understanding of faculty experiences. Furthermore, expanding the study at the European level would allow for the analysis of similarities and differences across higher education systems and advance the development of evidence-based occupational health policies tailored to the specific needs of health sciences university faculty.

## Figures and Tables

**Table 1:** Sociodemographic and professional characteristics of the sample (N = 253).

Variables	Mean (SD)/N (%)
Age	46.08 (11.48)
Gender	
Male Female Prefer not to say Other	80 (31.6%) 171 (67.6%) 1 (0.4%) 1 (0.4%)
Marital status	
Single Domestic partnership Married Prefer not to say Other	74 (20.2%) 20 (7.9%) 151 (59.7%) 7 (2.8%) 1 (0.4%)
Children	
None One child Two children Large family Prefer not to say	98 (38.7%) 50 (19.8%) 81 (32.0%) 20 (7.9%) 1 (0.4%)
Type of employment contract	
Part-time employee Part-time employee; self-employed Full-time employee Full-time employee; self-employed Self-employed Other	15 (5.9%) 4 (1.6%) 223 (88.1%) 3 (1.2%) 3 (1.2%) 5 (2.0%)
Academic position *	
Associate lecturer Lecturer Permanent Lecturer Senior Lecturer Full Professor Other positions (Substitute Lecturer, Predoctoral Researcher, collaborating lecturer)	58 (22.9%) 43 (17.0%) 41 (16.2%) 63 (24.9%) 15 (5.9%) 30 (11.9%)
Type of institution	
Private Public Both	33 (13.0%) 210 (83.0%) 10 (3.9%)
Disciplinary area	
Physiotherapy Occupational Therapy Psychology Medicine Dentistry Nursing Pharmacology Postgraduate education Other specialized training Multiple disciplines	36 (14.2%) 17 (6.7%) 17 (6.7%) 23 (9.1%) 7 (2.8%) 53 (20.9%) 1 (0.4%) 3 (1.2%) 22 (8.7%) 74 (29.2%)
Years of teaching experience	
6 months to less than 1 year 1 to less than 3 years 3 to less than 5 years 5 to less than 10 years 10 years or more	5 (2.0%) 41 (16.2%) 34 (13.4%) 53 (20.9%) 120 (47.4%)

Note: SD: standard deviation. Continuous variables are presented as mean ± standard deviation, and categorical variables are presented as n (%); * In Spain, Associate Lecturers are part-time professors, often combining teaching with professional work; Lecturers are early-career PhD holders focused on teaching and some research; Permanent Lecturers are tenured university staff involved in teaching, research, and academic duties; Senior Lecturers are experienced professors with stable positions, recognized for teaching and research; Full Professor is the highest rank, nationally accredited, leading in teaching, research, and academic management.

**Table 2:** Descriptive results of the psychosocial variables.

Variable (Scale)	Mean ± SD	Category Distribution
Perceived Stress (PSS-10)	15.5 ± 7.43	Low 41.1%; Moderate 49%; High 9.9%
Occupational Balance (OBQ-E)	42.5 ± 12.2	-
Professional Quality of Life (ProQOL)		
Compassion satisfaction	41.6 ± 6.5	Low 1.6%; Moderate 45.1%; High 53.4%
Burnout	24.9 ± 9.5	Low 48.2%; Moderate 46.2%; High 5.5%
Compassion fatigue	26.5 ± 6	Low 24.9%; Moderate 74.3%; High 0.8%
Occupational Satisfaction		
Work performance satisfaction	7.6 ± 1.5	-
Interpersonal satisfaction	6.1 ± 2.1	-
Satisfaction with work–rest–self-care balance	5.8 ± 2.3	-
Sense of Coherence (SOC-13)	67.1 ± 11.2	
Perceived Institutional Support		
Institutional concern for faculty well-being	2.58 ± 1.06	-
Work–life conciliation measures	2.74 ± 1.11	-
Support for emotional or professional difficulties	2.67 ± 1.06	-
Collaborative and respectful work climate	3.29 ± 1.30	-
Institutional mental health resources	2.58 ± 1.05	-
Autonomy in teaching and research tasks	3.66 ± 1.15	-
Opportunities to express professional concerns	2.80 ± 1.03	-
Consideration of actual working conditions	2.34 ± 0.99	-
Realistic and manageable workload	2.93 ± 1.24	-
Institutional recognition of teaching work	3.07 ± 1.18	-

Note: SD: standard deviation.

**Table 3:** Statistically significant associations and group differences between sociodemographic/professional variables and study constructs.

Psychosocial Construct	Significant Variable	Direction of the Effect/Group with Higher Score	Statistics
Perceived Stress (PSS-10)	Gender	Women > Men	F = 3.902; *p* = 0.008; d = 0.045
	Age	Negative relationship ( < stress with > age)	r = −0.229; *p* < 0.001; r^2^ = 0.05
	Academic position	Lecturer and permanent lecturer show higher values	F = 2.983; *p* = 0.012; η^2^ =0.058
	Years of teaching experience	Less and intermediate experience > more than 10 years	F = 3.050; *p* = 0.018; η^2^ = 0.047
Occupational Balance (OBQ-E)	Gender	Men > Women	F = 0.171; *p* = 0.020; d = 0.315
	Age	Positive relationship (> work–life balance with > age)	r = 0.191; *p* = 0.002; r^2^ = 0.04
	Marital status	Married individuals report higher levels	F = 3.532; *p* = 0.008; η^2^ = 0.054
	Academic position	“Other position” > remaining categories	F = 3.328; *p* = 0.006; η^2^ = 0.064
	Years of teaching experience	More than 10 years of experience associated with greater balance	F = 3.080; *p* = 0.017; η^2^ = 0.047
Professional Quality of Life (ProQoL)—Compassion satisfaction	Marital status	Cohabiting partners = lowest; married = highest	F = 7.174; *p* < 0.001; η^2^ = 0.104
	Academic position	Permanent lecturer = lowest	F = 3.946; *p* = 0.002; η^2^ = 0.075
	Type of institution	Public institutions > Private institutions	F = 3.718; *p* = 0.026; η^2^ = 0.029
Professional Quality of Life (ProQoL)—Burnout	Gender	Women > Men	F = 0.273; *p* = 0.030; d = 0.293
	Age	Negative relationship (< burnout with > age)	r = -0.219; *p* < 0.001; r^2^ = 0.048
Professional Quality of Life (ProQoL)—Compassion fatigue	Marital status	Cohabiting partners show higher scores	F = 4.026; *p* = 0.004; η^2^ = 0.061
Job performance satisfaction	Academic position	Full professor and “other position” show higher scores	F = 5.324; *p* < 0.001; η^2^ = 0.098
Interpersonal satisfaction	Age	Positive relationship (> satisfaction with > age)	r = 0.125; *p* = 0.049; r^2^ = 0.016
	Academic position	Senior academic positions > junior ranks	F = 3.081; *p* = 0.010; η^2^ = 0.060
Satisfaction with work–rest–self-care balance	Gender	Men > Women	F = 0.029; *p* = 0.005; d = 0.383
	Age	Positive relationship (> satisfaction with > age)	r = 0.187; *p* = 0.003; r^2^ = 0.035
Sense of coherence (SOC-13)	Academic position	Associate lecturer shows higher values	F = 2.707; *p* = 0.021; η^2^ = 0.053
Institutional Support—Concern for faculty well-being	Type of institution	Public institutions > Private institutions	F = 3.139; *p* = 0.045; η^2^ = 0.025
Institutional Support—Work–life conciliation measures	Type of institution	Public institutions > Private institutions	F = 3.216; *p* = 0.042; η^2^ = 0.025
Institutional Support—Support when facing emotional or professional difficulties	Type of institution	Public institutions > Private institutions	F = 7.918; *p* < 0.001; η^2^ = 0.060
Institutional Support—Collaborative and respectful work climate	Type of institution	Public institutions > Private institutions	F = 11.941; *p* < 0.001; η^2^ = 0.088
Institutional Support—Mental health resources	Academic position	Full professor reports higher scores	F = 2.268; *p* = 0.048; η^2^ = 0.044
Institutional Support—Autonomy in teaching and research tasks	Age	Positive relationship (> autonomy with > age)	r = 0.152; *p* = 0.016; r^2^ = 0.023
	Academic position	Senior academic positions exhibit greater autonomy	F = 2.256; *p* = 0.050; η^2^ = 0.044
Institutional Support—Opportunities to express professional concerns	Type of institution	Public institutions > Private institutions	F = 3.942; *p* = 0.021; η^2^ = 0.031
Institutional Support—Consideration of real working conditions	Academic position	Full professor = highest	F = 2.573; *p* = 0.027; η^2^ = 0.050

Note: This table presents only sociodemographic and professional variables showing statistically significant associations (Pearson correlations) or group differences (t-tests or ANOVA) with the evaluated constructs. Non-significant results are not displayed for clarity. For perceived institutional support, results are presented at the item level, as no aggregated score was calculated. Effect sizes are reported as Cohen’s *d* for gender comparisons, eta squared (η^2^) for ANOVA tests, and *r^2^* for correlation analyses.

## Data Availability

The data presented in this study are available on reasonable request from the corresponding author. The data are not publicly available due to ethical and privacy restrictions.

## Supplementary Materials

The following supporting information can be downloaded at https://www.mdpi.com/article/10.3390/healthcare14040494/s1: Table S1: STROBE checklist for cross-sectional studies. Table S2: Complete results of the associations between sociodemographic and professional variables and psychosocial study constructs, including significant and non-significant findings.

## References

[1] Matulevicius S.A., Kho K.A., Reisch J., Yin H. (2021). Academic Medicine Faculty Perceptions of Work-Life Balance Before and Since the COVID-19 Pandemic. JAMA Netw. Open.

[2] Asensio-Martínez Á., Aguilar-Latorre A., Masluk B., León-Herrera S., Recio R.S., Oliván-Blázquez B. (2026). Psychosocial and occupational predictors of health among Spanish University Employees: The role of job type, contract status, and seniority. PLoS ONE.

[3] Tello M.E.C., Boraita R.J., Ibort E.G., Torres J.M.D. (2025). Burnout Syndrome in Spanish University Lecturers: Prevalence and Relationship with Lifestyle Habits and Physical and Mental Health Indicators. BMC Psychol..

[4] Chavarría Islas R.A., Colunga Gutiérrez F.J., Loria Castellanos J., Peláez Méndez K. (2017). Síndrome de Burnout En Médicos Docentes de Un Hospital de 2. Nivel En México. Educ. Méd..

[5] Bernales-Turpo D., Quispe-Velasquez R., Flores-Ticona D., Saintila J., Ruiz Mamani P.G., Huancahuire-Vega S., Morales-García M., Morales-García W.C. (2022). Burnout, Professional Self-Efficacy, and Life Satisfaction as Predictors of Job Performance in Health Care Workers: The Mediating Role of Work Engagement. J. Prim. Care Community Health.

[6] López-Armendáriz K., González-Corzo I.G. (2024). Teletrabajo En La Pandemia Por COVID-19: Análisis de La Salud Mental y Factores Psicosociales de Académicos Universitarios Mexicanos. Acciones Investig. Soc..

[7] O’Meara K., Kuvaeva A., Nyunt G., Waugaman C., Jackson R. (2017). Asked More Often: Gender Differences in Faculty Workload in Research Universities and the Work Interactions That Shape Them. Am. Educ. Res. J..

[8] Misra J., Lundquist J.H., Templer A. (2012). Gender, Work Time, and Care Responsibilities Among Faculty. Sociol. Forum.

[9] Redondo-Flórez L., Tornero-Aguilera J.F., Ramos-Campo D.J., Clemente-Suárez V.J. (2020). Gender Differences in Stress- and Burnout-Related Factors of University Professors. Biomed Res. Int..

[10] Sabagh Z., Hall N.C., Saroyan A. (2018). Antecedents, Correlates and Consequences of Faculty Burnout. Educ. Res..

[11] Rojas-Solís J.L., Totolhua-Reyes B.A., Rodríguez-Vásquez D.J. (2021). Síndrome de Burnout En Docentes Universitarios Latinoamericanos: Una Revisión Sistemática. Rev. Espiral. Cuad. Profr..

[12] Deriglazov D., Halamová J., Kernová L. (2025). Burnout, Compassion Fatigue, and Compassion Satisfaction Interventions via Mobile Applications: A Systematic Review and a Meta-Analysis. Worldviews Evid. Based. Nurs..

[13] Beutel T., Koestner C., Wild P.S., Münzel T., Beutel M.E., Lackner K.J., Pfeiffer N., Nübling M., Becker J., Letzel S. (2023). Burnout, Self-Rated General Health and Life Satisfaction among Teachers and Other Academic Occupational Groups. Front. Public Health.

[14] Li H., Zuo M., Gelb A.W., Zhang B., Zhao X., Yao D., Xia D., Huang Y. (2018). Chinese Anesthesiologists Have High Burnout and Low Job Satisfaction: A Cross-Sectional Survey. Anesth. Analg..

[15] Varela J.J., Guzmán P., Oriol X., Romo F., Miranda R. (2023). Teachers’ Wellbeing, Affects, and Burnout during the Pandemic in Chile. Rev. Psicodidáctica.

[16] Bachman S., Cleveland P., Dale L., Santurri L. (2024). Entry-Level Occupational Therapy Student Perceptions of Occupational Balance in Graduate School: A Qualitative Study. J. Occup. Ther. Educ..

[17] Ramos R., Röschel A., Crevenna R., Jordakieva G., Andrews M.R., Dür M., Stamm T. (2022). Occupational Balance and Depressive Symptoms During the COVID-19 Pandemic. J. Occup. Environ. Med..

[18] Wagman P., Håkansson C. (2014). Introducing the Occupational Balance Questionnaire (OBQ). Scand. J. Occup. Ther..

[19] Huertas-Hoyas E., García-Bravo C., Pérez-Corrales J., Bullón-Benito E., Fernández-Gómez G., Rodríguez-Pérez M.P. (2025). Association between Occupational Balance and the Physical and Mental Health of the University Community: An Observational Study. Front. Public Health.

[20] Lehmann A.I., Bauer G.F. (2025). Occupational Health of Employees with Mental Health Issues: The Role of the Psychosocial Working Conditions and Sense of Coherence. Int. Arch. Occup. Environ. Health.

[21] Broetje S., Bauer G.F., Jenny G.J. (2020). The Relationship between Resourceful Working Conditions, Work-Related and General Sense of Coherence. Health Promot. Int..

[22] Aguilar-Latorre A., Asensio-Martínez Á., Oliván-Blázquez B., Álvarez-Bueno C., Cavero-Redondo I., Lionis C., Symvoulakis E.K., Magallón-Botaya R. (2023). Association between Sense of Coherence and Depression in Patients with Chronic Pain: A Systematic Review and Meta-Analysis. PLoS ONE.

[23] Geraci A., Di Domenico L., Inguglia C., D’Amico A. (2023). Teachers’ Emotional Intelligence, Burnout, Work Engagement, and Self-Efficacy during COVID-19 Lockdown. Behav. Sci..

[24] von Elm E., Altman D.G., Egger M., Pocock S.J., Gøtzsche P.C., Vandenbroucke J.P. (2014). The Strengthening the Reporting of Observational Studies in Epidemiology (STROBE) Statement: Guidelines for Reporting Observational Studies. Int. J. Surg..

[25] Remor E. (2006). Psychometric Properties of a European Spanish Version of the Perceived Stress Scale (PSS). Span. J. Psychol..

[26] Peral-Gómez P., López-Roig S., Pastor-Mira M.Á., Abad-Navarro E., Valera-Gran D., Håkansson C., Wagman P. (2021). Cultural Adaptation and Psychometric Properties of the Spanish Version of the Occupational Balance Questionnaire: An Instrument for Occupation-Based Research. Int. J. Environ. Res. Public Health.

[27] Parker D.M., Sykes C.H. (2006). A Systematic Review of the Canadian Occupational Performance Measure: A Clinical Practice Perspective. Br. J. Occup. Ther..

[28] Galiana L., Arena F., Oliver A., Sansó N., Benito E. (2017). Compassion Satisfaction, Compassion Fatigue, and Burnout in Spain and Brazil: ProQOL Validation and Cross-Cultural Diagnosis. J. Pain Symptom Manag..

[29] Vega Martínez M.d.C., Frías Osuna A., Del Pino Casado R. (2019). Validez y Confiabilidad de La Escala de Sentido de Coherencia En Estudiantes de Grado de Enfermería de Una Universidad Española. Gac. Sanit..

[30] Norman G. (2010). Likert Scales, Levels of Measurement and the “Laws” of Statistics. Adv. Health Sci. Educ..

[31] Lee M., Coutts R., Fielden J., Hutchinson M., Lakeman R., Mathisen B., Nasrawi D., Phillips N. (2022). Occupational Stress in University Academics in Australia and New Zealand. J. High. Educ. Policy Manag..

[32] González-Román L., Peral-Gómez P., Garrido-Pedrosa J., Zango-Martín I., Wagman P., Sánchez-Pérez A. (2023). Occupational Balance of Spanish Occupational Therapists—A Challenge. Scand. J. Occup. Ther..

[33] Sahni S., Kaushal L.A., Gupta P. (2025). Gendered Differences and Strategies for Work-Life Balance: Systematic Review Based on Social Ecological Framework Perspective. Acta Psychol..

[34] Giglio R.E., Rodriguez-Blazquez C., de Pedro-Cuesta J., Forjaz M.J. (2015). Sense of Coherence and Health of Community-Dwelling Older Adults in Spain. Int. Psychogeriatr..

[35] Instituto Nacional de Seguridad y Salud en el Trabajo (INSST), O.A., M.P Seguridad y Salud En El Trabajo. Estrategia Española, 2023–2027; Madrid, Spain, 2023. https://www.insst.es/documentacion/material-tecnico/documentos-tecnicos/estrategia-espa%C3%B1ola-de-seguridad-y-salud-en-el-trabajo-2023-2027.

