# Supplementary material for: Perceived Stress, Burnout, Professional Quality of Life, and Occupational Balance Among University Faculty in Health Sciences Disciplines in Spain—Protocol and Descriptive Results
Source: Healthcare (Basel). 2026 Feb 14;14(4):494. doi: 10.3390/healthcare14040494 (PMC12940884; doi:10.3390/healthcare14040494)
Supplement: Supplementary file 1 [file healthcare-14-00494-s001.zip › healthcare-4094251-supplementary.pdf]

Supplementary Material

Table S1. STROBE Checklist

STROBE Checklist for Cross-Sectional Studies

This supplementary file includes the completed STROBE (Strengthening the Reporting of Observational Studies in Epidemiology) checklist corresponding to the manuscript submitted for review.

STROBE Item	Recommendation	Section in Manuscript
1	Indicate the study design in title/abstract; informative abstract.	Title; Abstract
2	Explain scientific background and rationale.	Introduction
3	State specific objectives or hypotheses.	Introduction – Objectives
4	Present key elements of study design early.	Methods – Study Design
5	Describe setting, locations, and dates.	Methods – Participants & Procedure
6	Give eligibility criteria and selection methods.	Methods – Participants
7	Define all variables clearly.	Methods – Variables
8	Detail sources of data and measurement methods.	Methods – Instruments
9	Describe efforts to address potential bias.	Methods – Design
10	Explain how study size was determined.	Methods – Sample Size
11	Explain handling of quantitative variables.	Methods – Statistical Analysis
12	Describe all statistical methods.	Methods – Statistical Analysis
13	Report numbers of individuals at each stage.	Results – Sample Description
14	Give characteristics of study participants.	Results – Sociodemographic Characteristics
15	Report outcome data for each variable.	Results – Emotional Well-being & Occupational Balance
16	Provide unadjusted/adjusted estimates if applicable.	Not applicable (preliminary phase)
17	Report other analyses done.	Not applicable (preliminary phase)
18	Summarize key results.	Discussion
19	Discuss limitations.	Strengths and Limitations
20	Give interpretation considering objectives, limitations.	Discussion
21	Discuss generalizability of results.	Discussion; Strengths and Limitations
22	Provide source of funding.	Funding Statement

Supplementary Material

Table S2. Complete results of the associations between sociodemographic and professional variables and psychosocial study constructs, including significant and non-significant findings.

Psychosocial construct	Significant variable	Statistics
Perceived Stress (PSS-10)	Gender	F = 3.902; p = 0.008; d = 0.045
	Age	r = -0.229; p < 0.001; r² = 0.05
	Marital status	F = 1.632; p = 0.167; η² = 0.026
	Children	F = 1.052; p = 0.388; η² = 0.021
	Type of employment contract	F = 0.893; p = 0.486; η² = 0.018
	Academic position	F = 2.983; p = 0.012; η² =0.058
	Type of institution	F = 2.482; p = 0.086; η² = 0.019
	Disciplinary area	F = 0.994; p = 0.445; η² = 0.036
	Years of teaching experience	F = 3.050; p = 0.018; η² = 0.047
Occupational Balance (OBQ-E)	Gender	F = 0.171; p = 0.020; d = 0.315
	Age	r = 0.191; p = 0.002; r² = 0.04
	Marital status	F = 3.532; p = 0.008; η² = 0.054
	Children	F = 0.828; p = 0.531; η² = 0.017
	Type of employment contract	F = 0.489; p = 0.789; η² = 0.009
	Academic position	F = 3.328; p = 0.006; η² = 0.064
	Type of institution	F = 1.394; p = 0.250; η² = 0.011
	Disciplinary area	F = 0.898; p = 0.528; η² = 0.032
	Years of teaching experience	F = 3.080; p = 0.017; η² = 0.047
Professional Quality of Life (ProQoL) - Compassion satisfaction	Gender	F = 0.701; p = 0.429; d = 0.107
	Age	r = 0.037; p = 0.554; r² = 0.001
	Marital status	F = 7.174; p < 0.001; η² = 0.104
	Children	F = 0.486; p = 0.786; η² = 0.009
	Type of employment contract	F = 1.408; p = 0.222; η² = 0.028
	Academic position	F = 3.946; p = 0.002; η² = 0.075
	Type of institution	F = 3.718; p = 0.026; η² = 0.029
	Disciplinary area	F = 1.393; p = 0.192; η² = 0.049
	Years of teaching experience	F = 0.781; p = 0.538; η² = 0.012
Professional Quality of Life (ProQoL) – Burnout	Gender	F = 0.273; p = 0.030; d = 0.293
	Age	r = -0.219; p < 0.001; r² = 0.048
	Marital status	F = 1.577; p = 0.181; η² = 0.025
	Children	F = 0.938; p = 0.457; η² = 0.019
	Type of employment contract	F = 0.758; p = 0.581; η² = 0.015
	Academic position	F = 1.332; p = 0.251; η² = 0.027
	Type of institution	F = 1.822; p = 0.164; η² = 0.014
	Disciplinary area	F = 0.964; p = 0.470; η² = 0.034
	Years of teaching experience	F = 2.973; p = 0.020; η² = 0.046
Professional Quality of Life (ProQoL) – Compassion fatigue	Gender	F = 0.118; p = 0.117; d = 0.212
	Age	r = -0.002; p = 0.981; r² < 0.001
	Marital status	F = 4.026; p = 0.004; η² = 0.061

Supplementary Material

	Children	F = 0.206; p = 0.960; η^2 = 0.004
	Type of employment contract	F = 0.468; p = 0.800; η^2 = 0.009
	Academic position	F = 0.256; p = 0.936; η^2 = 0.005
	Type of institution	F = 1.630; p = 0.198; η^2 = 0.013
	Disciplinary area	F = 0.939; p = 0.491; η^2 = 0.034
	Years of teaching experience	F = 1.246; p = 0.292; η^2 = 0.019
Job performance satisfaction	Gender	F = 1.015; p = 0.103; d = 0.221
	Age	r = 0.090; p = 0.156; r^2 = 0.008
	Marital status	F = 0.011; p = 0.998; η^2 < 0.001
	Children	F = 1.294; p = 0.267; η^2 = 0.026
	Type of employment contract	F = 0.653; p = 0.660; η^2 = 0.013
	Academic position	F = 5.324; p < 0.001; η^2 = 0.098
	Type of institution	F = 1.287; p = 0.278; η^2 = 0.010
	Disciplinary area	F = 1.011; p = 0.432; η^2 = 0.037
	Years of teaching experience	F = 1.028; p = 0.393; η^2 = 0.017
Interpersonal satisfaction	Gender	F = 0.630; p = 0.190; d = 0.179
	Age	r = 0.125; p = 0.049; r^2 = 0.016
	Marital status	F = 0.028; p = 0.994; η^2 < 0.001
	Children	F = 0.559; p = 0.731; η^2 = 0.011
	Type of employment contract	F = 0.358; p = 0.877; η^2 = 0.007
	Academic position	F = 3.081; p = 0.010; η^2 = 0.060
	Type of institution	F = 1.126; p = 0.326; η^2 = 0.009
	Disciplinary area	F = 0.679; p = 0.728; η^2 = 0.025
	Years of teaching experience	F = 0.866; p = 0.485; η^2 = 0.014
Satisfaction with work-rest-self-care balance	Gender	F = 0.029; p = 0.005; d = 0.383
	Age	r = 0.187; p = 0.003; r^2 = 0.035
	Marital status	F = 0.424; p = 0.736; η^2 = 0.005
	Children	F = 0.613; p = 0.690; η^2 = 0.012
	Type of employment contract	F = 0.690; p = 0.631; η^2 = 0.014
	Academic position	F = 1.018; p = 0.408; η^2 = 0.020
	Type of institution	F = 1.711; p = 0.183; η^2 = 0.014
	Disciplinary area	F = 0.495; p = 0.877; η^2 = 0.018
	Years of teaching experience	F = 1.498; p = 0.203; η^2 = 0.024
Sense of coherence (SOC-13)	Gender	F = 0.563; p = 0.275; d = 0.147
	Age	r = 0.076; p = 0.229; r^2 = 0.052
	Marital status	F = 1.681; p = 0.172; η^2 = 0.020
	Children	F = 1.237; p = 0.292; η^2 = 0.025
	Type of employment contract	F = 0.906; p = 0.478; η^2 = 0.018
	Academic position	F = 2.707; p = 0.021; η^2 = 0.053
	Type of institution	F = 3.139; p = 0.045; η^2 = 0.025
	Disciplinary area	F = 0.938; p = 0.493; η^2 = 0.034
	Years of teaching experience	F = 1.231; p = 0.298; η^2 = 0.020
	Gender	F = 2.562; p = 0.257; d = 0.156

Supplementary Material

Institutional Support – Concern for faculty well-being	Age	r = 0.040; p = 0.532; r^2 = 0.002
	Marital status	F = 0.851; p = 0.467; η^2 = 0.010
	Children	F = 0.198; p = 0.963; η^2 = 0.004
	Type of employment contract	F = 0.985; p = 0.427; η^2 = 0.020
	Academic position	F = 1.206; p = 0.307; η^2 = 0.024
	Type of institution	F = 3.139; p = 0.045; η^2 = 0.025
	Disciplinary area	F = 1.068; p = 0.387; η^2 = 0.039
	Years of teaching experience	F = 1.731; p = 0.144; η^2 = 0.027
Institutional Support - Work-life conciliation measures	Gender	F = 1.769; p = 0.599; d = 0.070
	Age	r = -0.038; p = 0.553; r^2 = 0.001
	Marital status	F = 2.083; p = 0.103; η^2 = 0.025
	Children	F = 0.387; p = 0.858; η^2 = 0.008
	Type of employment contract	F = 0.317; p = 0.902; η^2 = 0.006
	Academic position	F = 1.362; p = 0.239; η^2 = 0.027
	Type of institution	F = 3.216; p = 0.042; η^2 = 0.025
	Disciplinary area	F = 0.686; p = 0.721; η^2 = 0.025
	Years of teaching experience	F = 1.934; p = 0.105; η^2 = 0.031
Institutional Support – Support when facing emotional or professional difficulties	Gender	F = 1.394; p = 0.570; d = 0.083
	Age	r = 0.013; p = 0.839; r^2 < 0.001
	Marital status	F = 0.981; p = 0.402; η^2 = 0.012
	Children	F = 0.160; p = 0.977; η^2 = 0.003
	Type of employment contract	F = 0.999; p = 0.419; η^2 = 0.020
	Academic position	F = 1.052; p = 0.388; η^2 = 0.021
	Type of institution	F = 7.918; p < 0.001; η^2 = 0.060
	Disciplinary area	F = 0.745; p = 0.668; η^2 = 0.027
	Years of teaching experience	F = 1.494; p = 0.204; η^2 = 0.024
Institutional Support – Collaborative and respectful work climate	Gender	F = 0.043; p = 0.132; d = 0.209
	Age	r = -0.059; p = 0.351; r^2 = 0.003
	Marital status	F = 0.791; p = 0.500; η^2 = 0.009
	Children	F = 0.247; p = 0.941; η^2 = 0.005
	Type of employment contract	F = 0.603; p = 0.697; η^2 = 0.012
	Academic position	F = 0.937; p = 0.458; η^2 = 0.019
	Type of institution	F = 11.941; p < 0.001; η^2 = 0.088
	Disciplinary area	F = 1.289; p = 0.244; η^2 = 0.046
	Years of teaching experience	F = 2.097; p = 0.082; η^2 = 0.033
Institutional Support – Mental health resources	Gender	F = 1.598; p = 0.953; d < 0.001
	Age	r = 0.089; p = 0.159; r^2 = 0.008
	Marital status	F = 0.869; p = 0.458; η^2 = 0.010
	Children	F = 0.651; p = 0.661; η^2 = 0.013
	Type of employment contract	F = 0.762; p = 0.578; η^2 = 0.015
	Academic position	F = 2.268; p = 0.048; η^2 = 0.044
	Type of institution	F = 2.618; p = 0.075; η^2 = 0.021
	Disciplinary area	F = 0.944; p = 0.488; η^2 = 0.034

Supplementary Material

	Years of teaching experience	$F = 1.400; p = 0.234; \eta^2 = 0.022$
Institutional Support - Autonomy in teaching and research tasks	Gender	$F = 0.006; p = 0.684; d = 0.052$
	Age	$r = 0.152; p = 0.016; r^2 = 0.023$
	Marital status	$F = 0.580; p = 0.629; \eta^2 = 0.007$
	Children	$F = 0.108; p = 0.991; \eta^2 = 0.002$
	Type of employment contract	$F = 1.269; p = 0.278; \eta^2 = 0.025$
	Academic position	$F = 2.256; p = 0.050; \eta^2 = 0.044$
	Type of institution	$F = 0.699; p = 0.498; \eta^2 = 0.006$
	Disciplinary area	$F = 1.140; p = 0.335; \eta^2 = 0.041$
	Years of teaching experience	$F = 0.950; p = 0.436; \eta^2 = 0.015$
Institutional Support - Opportunities to express professional concerns	Gender	$F = 2.668; p = 0.083; d = 0.226$
	Age	$r = 0.069; p = 0.280; r^2 = 0.005$
	Marital status	$F = 0.292; p = 0.831; \eta^2 = 0.004$
	Children	$F = 0.538; p = 0.747; \eta^2 = 0.011$
	Type of employment contract	$F = 0.564; p = 0.727; \eta^2 = 0.011$
	Academic position	$F = 0.688; p = 0.633; \eta^2 = 0.014$
	Type of institution	$F = 3.942; p = 0.021; \eta^2 = 0.031$
	Disciplinary area	$F = 1.177; p = 0.310; \eta^2 = 0.042$
	Years of teaching experience	$F = 1.239; p = 0.295; \eta^2 = 0.020$
Institutional Support - Consideration of real working conditions	Gender	$F = 1.508; p = 0.774; d = 0.040$
	Age	$r = 0.035; p = 0.577; r^2 = 0.001$
	Marital status	$F = 0.423; p = 0.737; \eta^2 = 0.005$
	Children	$F = 0.815; p = 0.540; \eta^2 = 0.016$
	Type of employment contract	$F = 0.325; p = 0.898; \eta^2 = 0.007$
	Academic position	$F = 2.573; p = 0.027; \eta^2 = 0.050$
	Type of institution	$F = 0.452; p = 0.637; \eta^2 = 0.004$
	Disciplinary area	$F = 1.090; p = 0.371; \eta^2 = 0.039$
	Years of teaching experience	$F = 0.942; p = 0.440; \eta^2 = 0.015$
Institutional Support - Realistic and manageable workload	Gender	$F = 1.201; p = 0.482; d = 0.092$
	Age	$r = 0.060; p = 0.347; r^2 = 0.004$
	Marital status	$F = 2.519; p = 0.059; \eta^2 = 0.030$
	Children	$F = 0.746; p = 0.589; \eta^2 = 0.015$
	Type of employment contract	$F = 1.828; p = 0.108; \eta^2 = 0.036$
	Academic position	$F = 1.680; p = 0.140; \eta^2 = 0.033$
	Type of institution	$F = 2.301; p = 0.102; \eta^2 = 0.018$
	Disciplinary area	$F = 0.790; p = 0.626; \eta^2 = 0.029$
	Years of teaching experience	$F = 1.608; p = 0.173; \eta^2 = 0.026$
	Gender	$F = 4.038; p = 0.432; d = 0.099$

Supplementary Material

Institutional Support - Institutional recognition of teaching work	Age	r = 0.045; p = 0.482; r^2 = 0.002
	Marital status	F = 0.516; p = 0.671; η^2 = 0.006
	Children	F = 0.567; p = 0.726; η^2 = 0.011
	Type of employment contract	F = 1.384; p = 0.231; η^2 = 0.028
	Academic position	F = 1.782; p = 0.117; η^2 = 0.035
	Type of institution	F = 2.135; p = 0.120; η^2 = 0.017
	Disciplinary area	F = 1.327; p = 0.223; η^2 = 0.047
	Years of teaching experience	F = 0.664; p = 0.618; η^2 = 0.011
